# Uev1A-Ubc13 promotes colorectal cancer metastasis through regulating *CXCL1* expression via NF-кB activation

**DOI:** 10.18632/oncotarget.24640

**Published:** 2018-03-23

**Authors:** Zhaojia Wu, Heather Neufeld, Eminao Torlakovic, Wei Xiao

**Affiliations:** ^1^ Department of Microbiology and Immunology, University of Saskatchewan, Saskatoon S7N 5E5, Canada; ^2^ Department of Pathology and Laboratory Medicine, University of Saskatchewan, Saskatoon S7N 5E5, Canada; ^3^ Current address: Department of Laboratory Hematology, Toronto General Hospital/UHN, Toronto M5G 2C4, Canada

**Keywords:** colorectal cancer, metastasis, Uev1A, NF-кB, *CXCL1*

## Abstract

Colorectal cancer is the second most common cause of cancer-related death worldwide. Uncontrolled growth and distant metastasis are hallmarks of colorectal cancer. However, the precise etiological factors and the mechanisms are diverse and still largely unclear. The potential proto-oncogene *UEV1A* encodes a ubiquitin conjugating enzyme variant, which is required for Ubc13-catalyzed K63-linked poly-ubiquitination of target proteins and the activation of NF-кB, a transcription factor known to be involved in innate immunity, anti-apoptosis, inflammation and cancer. In order to understand the roles of Uev1A in colon cancer progression, we experimentally manipulated the Uev1A level in HCT116 colon cancer cells and found that *UEV1A* overexpression alone is sufficient to promote invasion *in vitro* and metastasis *in vivo*. This process is mediated by NF-κB activation and depends on its physical interaction with Ubc13. No expression of Uev1A was detected in histologically normal human colonic mucosa, but its expression was detected in human colorectal adenocarcinoma, which was closely correlated with nuclear p65 levels, an indicator of NF-κB activation. Uev1A protein was detected in 46% of primary tumors and 79% of metastatic tumors examined. Our experimental data establish that among NF-κB target genes, Uev1A-regulated *CXCL1* expression plays a critical role in colon cell invasion and metastasis, a notion supported by the colon adenocarcinoma survey. Furthermore, experimental depletion of Uev1 in HCT116 cells reduces *CXCL1* expression, and prevents cell invasion and tumor growth in a xenograft mouse model. These results identify Uev1A as a potential therapeutic target in the treatment of metastatic colorectal cancers.

## INTRODUCTION

*UEV1* (also known as *CROC1* or *CIR1*), encoding a ubiquitin (Ub)-conjugating enzyme variant (Uev), was identified as a mammalian homolog of yeast *MMS2* [1], as well as a gene whose expression was positively corelated to tumorigenesis in various screens [2–4]. A Uev (Uev1 or Mms2) is a cofactor and absolutely required for Ubc13-mediated K63-linked polyubiquitin chain assembly [5–8]. *UEV1* encodes at least three splicing variants, among which Uev1A and Uev1C are able to form a complex with Ubc13 and promote K63-linked polyubiquitination but differ in that Uev1A contains thirty additional amino acids at the N-terminus [9, 10].

Although Uev1A and Mms2 have similar biochemical activity, they appear to function differently in mammalian cells: Ubc13-Mms2 is required for DNA-damage response whereas Ubc13–Uev1A is involved in NF-κB activation [10]. The Uev1A-Ubc13 heterodimer acts downstream of tumor-necrosis factor (TNF) receptors along with TNF-associated factor 6 (TRAF6) [11, 12] and TRAF2 [13] to polyubiquitinate NEMO/IKKγ [14, 15] and/or RIP1 [16] to activate IKK. Activated IKK leads to the phosphorylation and degradation of IκBα, resulting in the release of NF-κB RelA (p65) subunits to translocate into the nucleus [17]. NF-кB is a sequence-specific transcription factor known to be involved in innate immunity, anti-apoptosis and inflammation [18–20], and its uncontrolled activation is associated with cancers [21, 22]. Previous studies reported that Ubc13-Uev1A inhibits stress-induced apoptosis in HepG2 cells [23] and overexpression of *UEV1A* in MDA-MB-231 cells is sufficient to induce metastasis both *in vitro* and *in vivo* primarily through regulating matrix metalloproteinase-1 (*MMP1*) gene expression [9]. Both functions are regulated by NF-κB activation and require functional Ubc13. These observations collectively establish a close correlation between *UEV1A* expression and tumorigenic potential.

In fact, many previous studies implicate *UEV1* as a potential proto-oncogene. *UEV1* maps to chromosome 20q13.2 [4], a region where DNA amplification is frequently reported in breast cancers [24–26] and other tumors [27], as well as in virus-transformed immortal cells [28]. *UEV1* was identified as a transactivator of the *c-fos* promoter [3]. It is down-regulated when HT29-M6 colon cancer cells undergo chemical-induced differentiation [4], and up-regulated when SV40-transformed human embryonic kidney cells become immortal [2]. Furthermore, *UEV1* is variably up-regulated in all tumor cell lines examined [1, 9]. It was reported that a small-molecule inhibitor of Ubc13-Uev1A interaction can inhibit proliferation and survival of diffuse large B-cell lymphoma cells [29]. Although elevated expression of *UEV1* has been reported in various human tumors, the role of *UEV1* in human colorectal cancers (CRCs) is poorly understood.

In this study we demonstrate that in HCT116 colon cancer cells, the *UEV1A* transcript level is moderately elevated compared to normal colon cells, that elevated Uev1A levels are frequently observed in primary and metastatic colon cancers, and that, more importantly, elevated Uev1A is highly correlated with the nuclear translocation of the p65 subunit of NF-κB in human colon cancer samples. Indeed, experimental overexpression of *UEV1A* alone in HCT116 cells is sufficient to activate NF-кB, which in turn up-regulates the *CXCL1* expression to enhance colon cancer cell metastasis. On the other hand, experimental depletion of Uev1 in HCT116 cells reduces *CXCL1* expression, cell invasion *in vitro* and the ability to grow tumors in a xenograft mouse model. These observations suggest a potential therapeutic target in the treatment of metastatic colon cancers.

## RESULTS

### *UEV1A* is overexpressed in colon cancers

**Figure 1:**
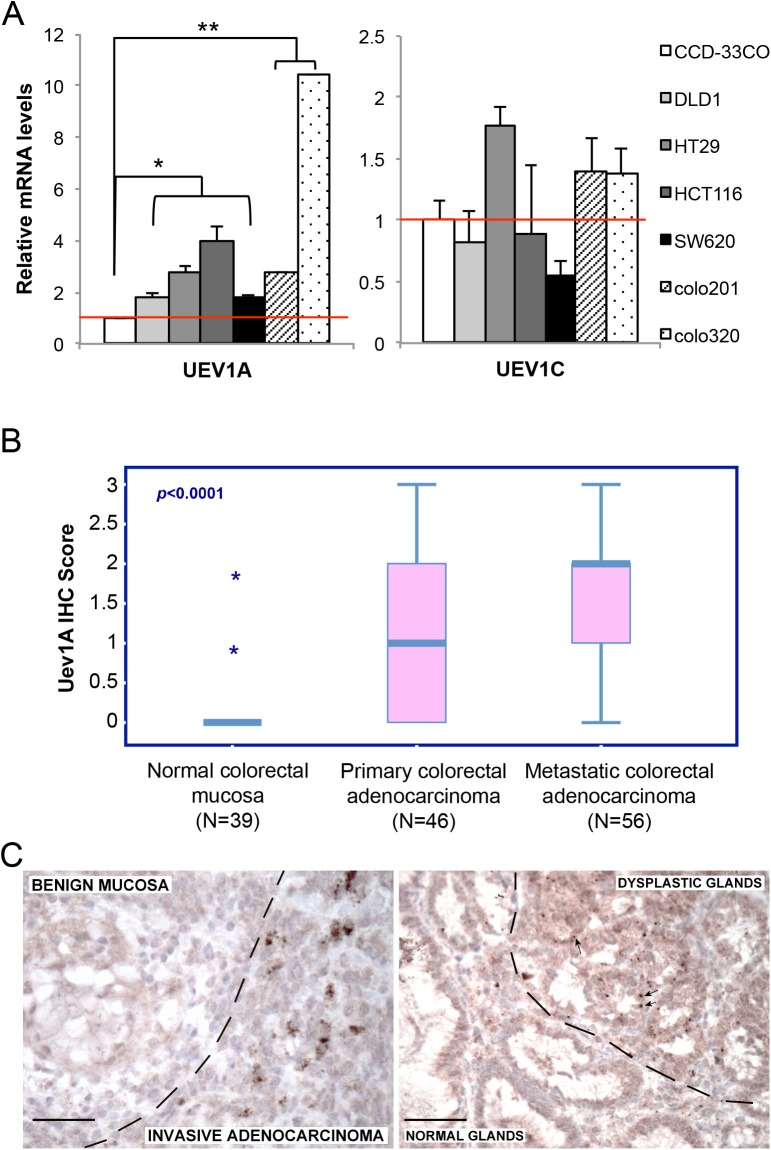
*UEV1A* is overexpressed in human colon cancer cell lines and tumor samples **(A)** Relative transcript levels of *UEV1A* and *UEV1C* variants in human colon cancer cell lines as determined by qRT-PCR.(^*^ *P*<0.05, ^**^ *P*<0.01) **(B)** Relative cellular Uev1A level as determined by immunohistochemistry using a Uev1A-specific antibody (LN1) against formalin-fixed/paraffin-embedded human colon carcinoma samples. **(C)** Representative IHC images containing both normal and tumor tissues showing differential LN1 staining. Dotted lines indicate border of normal and tumor tissues. Scale bar = 50 μm.

To investigate the role of *UEV1A* in human colon cancers, we examined relative transcript levels of *UEV1A* and *UEV1C* in colon cancer lines using human normal colon cell CCD-33Co as a reference. Interestingly, the *UEV1A* transcript level is elevated in all colon cancer cell lines examined (Figure 1A, left), with no more than twofold upregulation of *UEV1C* in these lines (Figure 1A, right).

It has been previously reported that elevated expression of *UEV1A* may promote tumor growth and metastasis in a breast tumor model [9]. We next assessed relative *UEV1A* expression in normal and malignant colon tumors by immunohistochemistry (IHC) using a Uev1A-specific monoclonal antibody LN1 against formalin-fixed/paraffin-embedded tissue samples, based on a blind scoring scale as shown in Supplementary Figure 1. In general, normal colonic mucosa does not express Uev1A, but its expression is frequently detected in human colon adenocarcinoma, while metastatic tumors showed significantly stronger staining for Uev1A than in primary colon adenocarcinoma (Pearson Chi-Square Test, *p<*0.001). For example, only 3/31 benign samples showed weak focal expression of Uev1A, while samples of primary colon carcinoma were positive in 21/46 (46%), and metastatic colon carcinoma was positive in 44/56 (79%) cases (Figure 1B). Of particular interest is that in occasional cases where both normal and tumorigenic tissues are present in the same patient sample, Uev1A expression is specifically observed in the tumorigenic tissues but not in normal tissues (Figure 1C). The above observations collectively indicate that *UEV1A* is frequently overexpressed in colon cancers.

### *UEV1A* regulates colon cancer cell invasion *in vitro* and metastasis in a xenograft model

**Figure 2:**
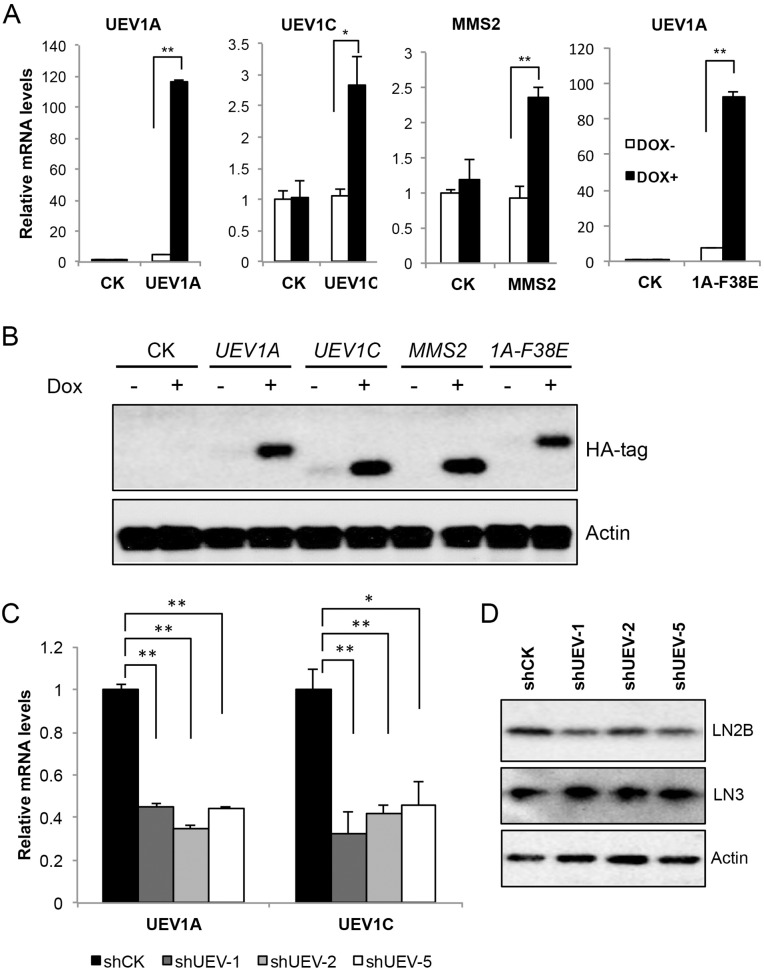
Manipulation of *UEV* expression levels in HCT116 cells cDNA4.0/TO/HA(+) vector expressing *UEV1A*, *UEV1C*, *MMS2,* mutated *UEV1A* (*1A-F38E*) or vector only (CK) was stably transfected into HCT116-TR cells, with or without Dox treatment. The level of ectopic gene expression was monitored **(A)** by qRT-PCR and **(B)** by western blot against an anti-HA antibody. **(C)** *UEV1A* and *UEV1C* transcript levels in shCK and shUEV1 lines were determined by qRT-PCR. HCT116 cells were transfected with shRNA lentiviral particles against *UEV1* (sh*UEV1*) or non-specific target (shCK). 20 single colonies were picked and subcultured. shUEV1-1, shUEV1-2 and shUEV1-5 represent three independent stable shUEV1 lines. **(D)** Uev1C and Mms2 levels in shCK and shUEV1 lines were determined by western blot against anti-Uev1 (LN2B) and MMS2+Uev1 (LN3) antibodies. ^*^ *P*<0.05, ^**^ *P*<0.01.

To ask whether an elevated *UEV1A* level alone is sufficient to promote colon tumorigenesis, *UEV1A*, *UEV1C* or *MMS2* genes were cloned into a pcDNA4.0/TO/HA(+) vector and then transfected into HCT116-TR cells to create stable cell lines, and the level of ecotopic gene expression after 10 μg/ml doxycycline (Dox) treatment was monitored by qRT-PCR (Figure 2A) and by western blot against the HA tag (Figure 2B). We also created a stable HCT116-TR cell line expressing Dox-inducible Uev1A-F38E mutant protein (Figure 2A and 2B) that abolishes its interaction with Ubc13 and thus its ability to promote Ubc13-mediated K63 polyubiquitination [8].

**Figure 3:**
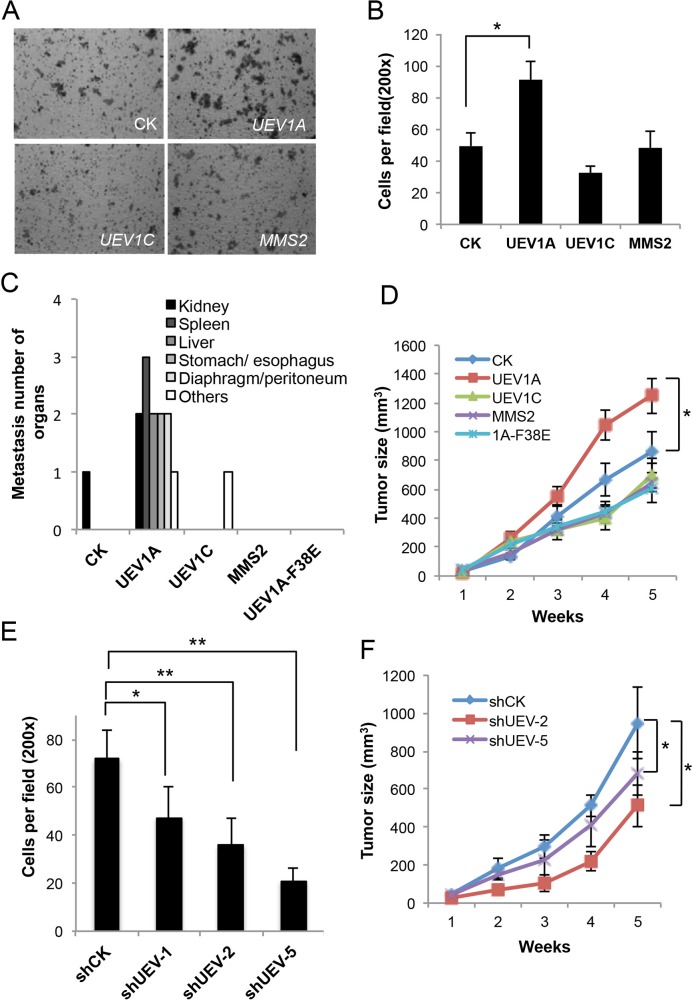
Experimental manipulation of *UEV1A* expression alters HCT116 cell invasion *in vitro* and metastasis in a xenograft mouse model **(A)** Representative images of cell invasion assay with Matrigel-coated transwells in vector control (CK), *UEV1A*, *UEV1C* or *MMS2* stably-transfected HCT116-TR cells with Dox treatment. **(B)** Statistical analysis of the cell invasion assay data. Cells that invaded the lower surface of the filter were counted in 5 random fields under a light microscope at 200x magnification (^*^ *P*<0.05). **(C)** *In vivo* tumorigenesis and metastasis assays using a xenograft mouse model. 1×10^6^ HCT116-TR cells collected from each treatment were injected into the lateral flanks of 4- to 5-week-old female athymic nude mice. Five weeks after injection, organ samples were taken after sacrifice. Quantitative analysis of the *in vivo* organ metastasis as measured by the number of metastatic organs (n = 5). **(D)** *In vivo* tumorigenesis assays using a xenograft mouse model. Tumor growth was measured every week after injection (Day 0) and expressed as mean ± SD (n = 10), (^*^ *P*<0.05). **(E)** Quantitative analysis of cell invasion in Matrigel-coated transwells. Cells that invaded the lower surface of the filter were counted in 5 random fields under a light microscope at 200x magnification (^*^ *P*<0.05, ^**^ *P*<0.01). **(F)** The *in vivo* tumorigenesis assay using a xenograft mouse model. 1×10^6^ HCT116 cells depleted with shUEV1 or shCK were injected into the lateral flanks of 4 to 5-week-old female athymic nude mice. Tumor growth was measured every week after injection (Day 0) and expressed as mean ± SD (n = 10), (^*^ *P*<0.05).

The cell growth of stable HCT116-TR transfectants was first measured, with no significant alterations among each group in 6 days (Supplementary Figure 2). The effects of ecotopic gene expression on colon cancer cell invasion were then measured. In a transwell assay, the invasiveness of *UEV1A* transfectants was approximately 1.85-fold higher than the control, *UEV1C* or *MMS2* transfectants, whereas there is no significant difference among control, *UEV1C* and *MMS2* transfectants (Figure 3A and 3B). Furthermore, we overexpressed *UEV1A* in another colon cancer cell line, DLD1 (Supplementary Figure 3A, 3B). The invasiveness of *UEV1A* transfectants was approximately 1.66-fold higher than the control in DLD1 cells as measured by the transwell assay (Supplementary Figure 7D). These results suggest that *UEV1A* but not *UEV1C* or *MMS2* promotes colon cancer cell invasion *in vitro*.

As an increased ability of cancer cells to invade *in vitro* may indicate an increased ability to cause cell metastasis, to further address the correlation between *UEV1A* expression and colon cancer metastasis, we assessed the effects of *UEV1A* on metastasis using an *in vivo* xenograft mouse model. Stably-transfected HCT116-TR cells were injected into the lateral flanks of 4- to 5-week-old female athymic nude mice and 625 mg/kg Doxycycline (Dox) was added in feed as soon as the cells were injected. Tumor growth and metastasis were then monitored. Compared to those injected with either vector, *UEV1C* or *MMS2*, the mice injected with the *UEV1A*-expressing cells had metastasis tumors on many organs, such as spleen, liver, kidney, stomach/esophagus and diaphragm/peritoneum (Figure 3C and Supplementary Figure 4). In contrast, there was no tumor metastasis in mice injected with *MMS2*-expressing cells or cells expressing mutated *UEV1A-F38E*. One of ten mice injected with vector control cells developed kidney metastasis and one of ten mice injected with *UEV1C*-expressing cells developed an metastasis tumor on shoulder. Furthermore, overexpression of *UEV1A* but not *UEV1C* or *MMS2,* accelerated tumor growth compared to vector-transfected cells (Figure 3D).

The endogenous *UEV1* expression in HCT116 cells was suppressed by using an shRNA designed against *UEV1* (shUEV1) delivered by lentiviral particles. 20 independent colonies were picked and individually subcultured. The expression levels of *UEV1A, UEV1C or MMS2* were determined by qRT-PCT. It was found that 3 shUEV1 colonies, shUEV1-1, shUEV1-2 and shUEV1-5, reduced *UEV1A* expression to 45%, 35%, and 44% of control shRNA-treated cells, respcetively (Figure 2C). As expected, the cellular *UEV1C* mRNA and protein levels were also reduced (Figure 2C and 2D) but the *MMS2* expression remains unaffected (Figures 2D and Supplementary Figure 5A).

Partial depletion of Uev1 reduced cell invasion (Figures 3E and Supplementary Figure 5B). The above findings were further extended by using a xenograft mouse model, in which depletion of Uev1 limited tumor growth (Figure 3F), and like the control group, no metastasis to any organs was observed (data not shown). These results collectively indicate that elevated *UEV1A* expression in HCT116 cells plays a critrical role in colon cancer tumorigenesis and metastasis.

### Overexpression of *UEV1A* activates NF-κB in colon cancer cells in a Ubc13-dependent manner

To understand the mechanism by which Uev1A promotes metastasis in colon cancer cells, we took into account that Uev1A has been reported to activate NF-κB in HepG2 cells [23], and that, once released into the nucleus, NF-κB regulates the expression of a large number of genes critical for tumorigenesis, inflammation and metastasis [21]. Our previous research also reveals that Uev1A can promote breast cancer metastasis though NF-κB activation [9]. IHC, which was performed in 102 colon tumor samples (Table 1A) and 56 normal tissue samples (Table 1B), reveals a close correlation between *UEV1A* overexpression and NF-κB activation as judged by the nuclear translocation of the p65 subunit of NF-κB (Spearman correlation, r=0.389, *p*<0.0001 for all tissues).

**Table 1A:** Correlation of cytoplasmic Uev1A levels with nuclear p65

Nuclear p65		Uev1A	(LN1)		Total
	0	1	2	3	
**0 (HS<10)**	9	2	2	0	13
**1 (HS=10-100)**	6	6	16	5	33
**2 (HS=101-200)**	7	12	15	6	40
**3 (HS=201-300)**	3	4	3	6	16
**Total**	25	24	36	17	102

Tumor samples only (*P* = 0.008).

**Table 1B:** Correlation of cytoplasmic Uev1A levels with nuclear p65

Nuclear p65		Uev1A	(LN1)		Total
	0	1	2	3	
**0**	37	8	1	0	46
**1**	3	3	1	1	8
**2**	1	1	0	0	2
**3**	0	0	0	0	0
**Total**	41	12	2	1	56

Normal tissues only (*P* < 0.001).

**Figure 4:**
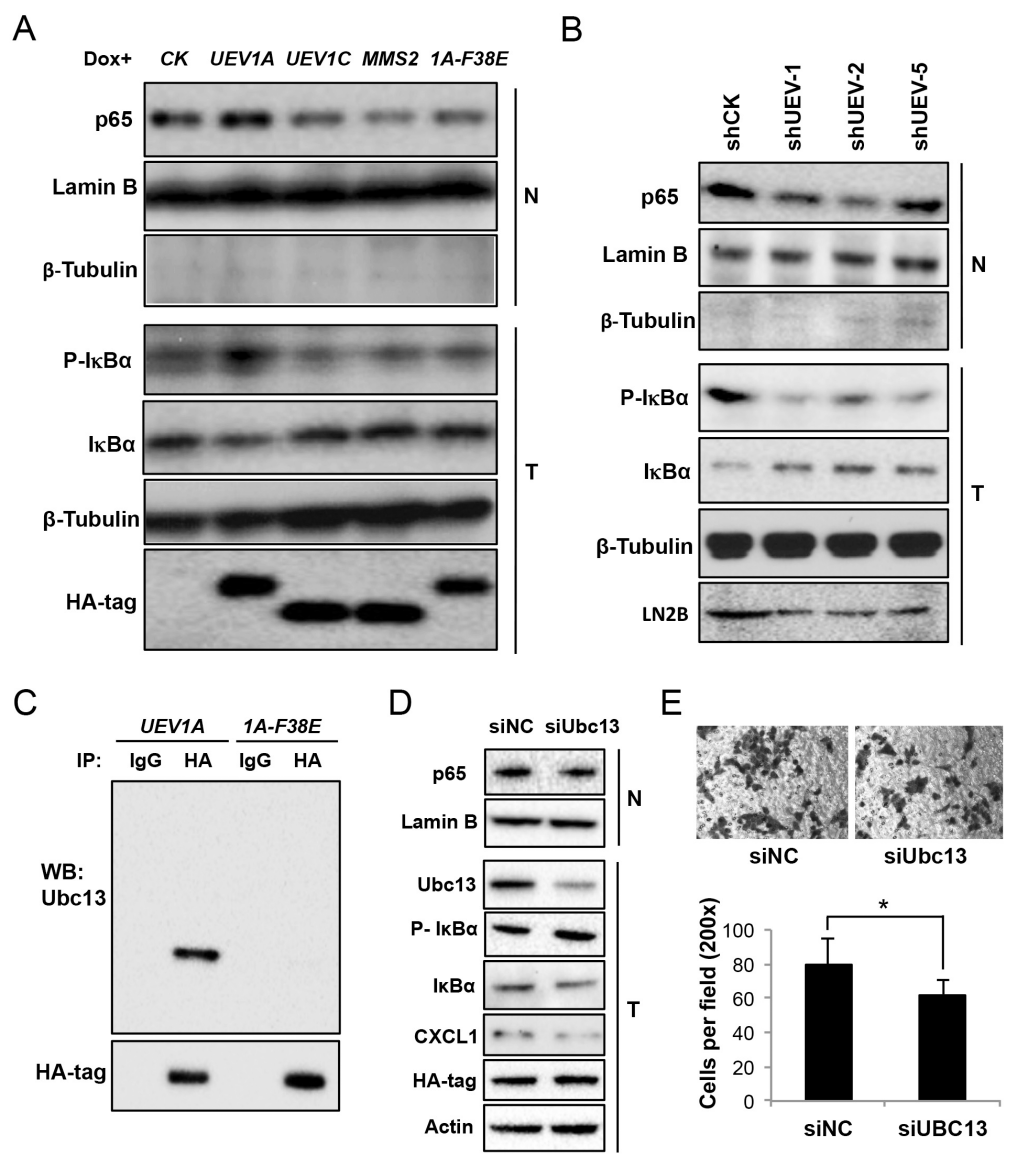
Uev1A activates NF-κB in colon tumors and HCT116 cells in a Ubc13-dependent manner **(A)** NF-κB activation in *UEV*-overexpressing cells. Nuclear (N) or whole-cell (T) extracts were prepared and equal amounts of protein were separated by SDS-PAGE gel, followed by western blotting analysis using an anti-p65 antibody to measure NF-кB nuclear enrichment, an anti-P(S32)-IκBα antibody and an anti-IκBα antibody to assess the degree of IκBα phosphorylation and its degradation and release of NF-κB into the nucleus. **(B)** NF-κB activation in Uev1-depleted cells. Experimental conditions are as described in (A). **(C)** Uev1A-F38E (1A-F38E) fails to interact with Ubc13 as judged by a co-IP assay. **(D)** NF-κB activation in Ubc13-depleted cells. Experimental conditions are as described in (A). **(E)** Cell invasion assay with Matrigel-coated transwells in Ubc13 depleted and *UEV1A* overexpressed cells. Top panel, representative images; lower panel, quantitative analysis (^*^ *P*<0.05).

Since a hallmark of NF-κB activation is its translocation from cytoplasm to the nucleus, we transfected HCT116-TR cells with a variety of constructs, induced the target gene expression by adding Dox, fractionated cells and then measured the subcellular distribution of the p65 subunit of NF-κB. As seen in Figure 4A, only overexpression of *UEV1A*, but not *UEV1C* or *MMS2*, was able to increase the phosphorylation of the NF-κB inhibitor IκBα, decrease cellular IκBα and enrich p65 in the nucleus. Consistently, depletion of Uev1 by shRNA reduced IκBα phosphorylation and p65 nuclear translocation (Figure 4B). Similar to HCT116, only overexression of *UEV1A*, but not *UEV1C* or *MMS2* was able to increase the phosphorylation of IκBα, decrease IκBα and promote more p65 into the nucleus in DLD1 cells (Supplementary Figure 3C).

It has been reported that Uev1A works together with Ubc13 as a E2 complex to promote K63-linked polyubiquitination of target proteins in the NF-κB signaling pathway [10, 11]. To ask whether Uev1A indeed requires Ubc13 for this process, we took two aproaches. First, a Mms2-F13E mutation is known to abolish the Mms2-Ubc13 complex formation [8], and similar mutations in Uev1A have been utilized to distinguish whether the Uev1 activity is dependent on Ubc13 [9, 30]. The Uev1A-F38E mutation completely abolished the physical interaction with Ubc13 *in vivo* (Figure 4C), and overexpression of such mutant gene failed to activate NF-κB (Figure 4A), indicating that the Uev1A-Ubc13 complex formation is absolutely required. Secondly, we attempted to deplete cellular Ubc13 in the *UEV1A* overexpressed cells to see whether limit of the Ubc13 level can reverse *UEV1A*-induced phenotypes. Indeed Ubc13 depletion increased phosphorylated IκBα, decreased total cellular IκBα and reduced nuclear p65 (Figure 4D), which led to decreased cell invasion (Figure 4E). Taken together, we conclude that *UEV1A* overexpression resulted in increased Uev1A-Ubc13 complex formation, which promotes NF-κB signaling.

### *CXCL1* and *MMP9* genes are regulated by *UEV1*

**Figure 5:**
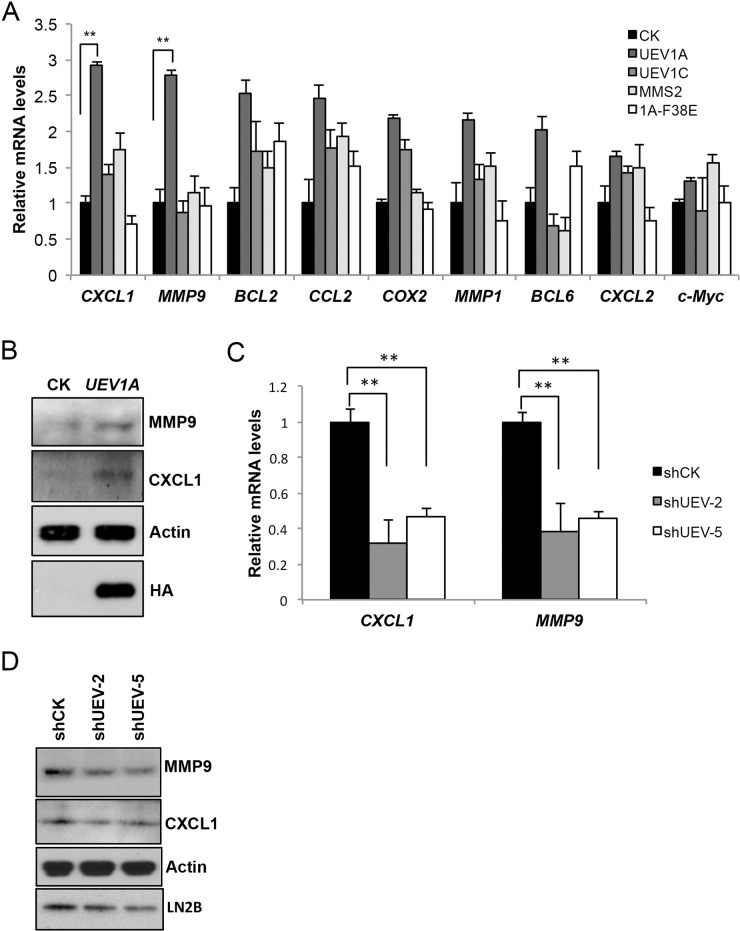
Uev1A positively regulates *CXCL1* and *MMP9* expression **(A)** Transcript levels of selected putative NF-κB target genes in HCT116-TR cells expressing different *UEV*s as determined by qRT-PCR. **(B)** Elevated CXCL1 and MMP9 protein levels in *UEV1A*-overexpressed HCT116 cells as determined by western blot. Actin serves as a loading control and HA-tag shows ectopic *UEV1A* overexpression. **(C)** Relative transcript levels of *CXCL1* and *MMP9* in two independent shUEV1-transfected HCT116 cell lines as determined by qRT-PCR. **(D)** Relative protein levels of *CXCL1* and *MMP9* in two independent shUEV1-transfected HCT116 cell lines as determined by western blot. ^**^ *P*<0.01.

Since NF-κB is a transcription factor that regulates the expression of a large number of genes including those involved in tumorigenesis and metastasis, we assessed transcript levels of many established or tentative NF-κB target genes thought to be involved in cancer such as *MMP1* [9], *MMP9* [31], *BCL2* [32], *BCL6* [33], *COX2* [34], *c-Myc* [35], *CCL2* [36] and *CXCL1* [37], among which *CXCL1 and MMP9* transcripts were elevated by 2.9- and 2.8-fold, respectively, in *UEV1A*-overexpression cells, but not in *UEV1C or MMS2* overexpression cells (Figure 5A). CXCL1 and MMP9 protein levels were also elevated after *UEV1A*-overexpression (Figure 5B). This effect is completely dependent on Ubc13, as overexpression of *UEV1A-F38E* failed to induce *CXCL1* and *MMP9* (Figure 5A). Similarly, depletion of Uev1 in HCT116 cells significantly reduced *CXCL1* and *MMP9* transcript (Figure 5C) and protein (Figure 5D) levels, indicating that *CXCL1* and *MMP9* are regulated by Uev1A-Ubc13. *CXCL1* and *MMP9* transcript levels were also elevated by 6.8-fold and 2.3-fold, respectively, in *UEV1A*-overexpressed DLD1 cells (Supplementary Figure 3D).

It has been previously reported that in MDA-MB-231 breast cancer cells, Uev1A upregulates *MMP1*, which is a dominant factor. *MMP1* can also be upregulated 2.2-fold in *UEV1A*-overexpressed HCT116-TR cells, but is not significantly different from levels in *UEV1C*- or *MMS2*-overexpressed cells (Figure 5A). To ask whether MMP1 and MMP9 are critical effectors for Uev1A-induced metastasis, we depleted MMP1 (Supplementary Figure 6A) or MMP9 (Supplementary Figure 6B) by siRNA in HCT116 cells. The transwell assay results (Supplementary Figure 6C) show that *MMP1* depletion reduces HCT116 cell invasion while *MMP9* depletion does not.

### CXCL1 is a downstream effector for Uev1A-induced metastasis in colon cancer cells

*CXCL1* encodeschemokine (C-X-C motif) ligand 1, also known as growth-regulated oncogene α (GRO) or melanoma growth stimulatory activity (MGSA). Chemokines are a group of small molecular cytokines that play important roles not only in the regulation of inflammation, wound healing, and development, but also in tumorigenesis and tumor metastasis through the G-protein-coupled receptor CXCR [37–43].

**Figure 6:**
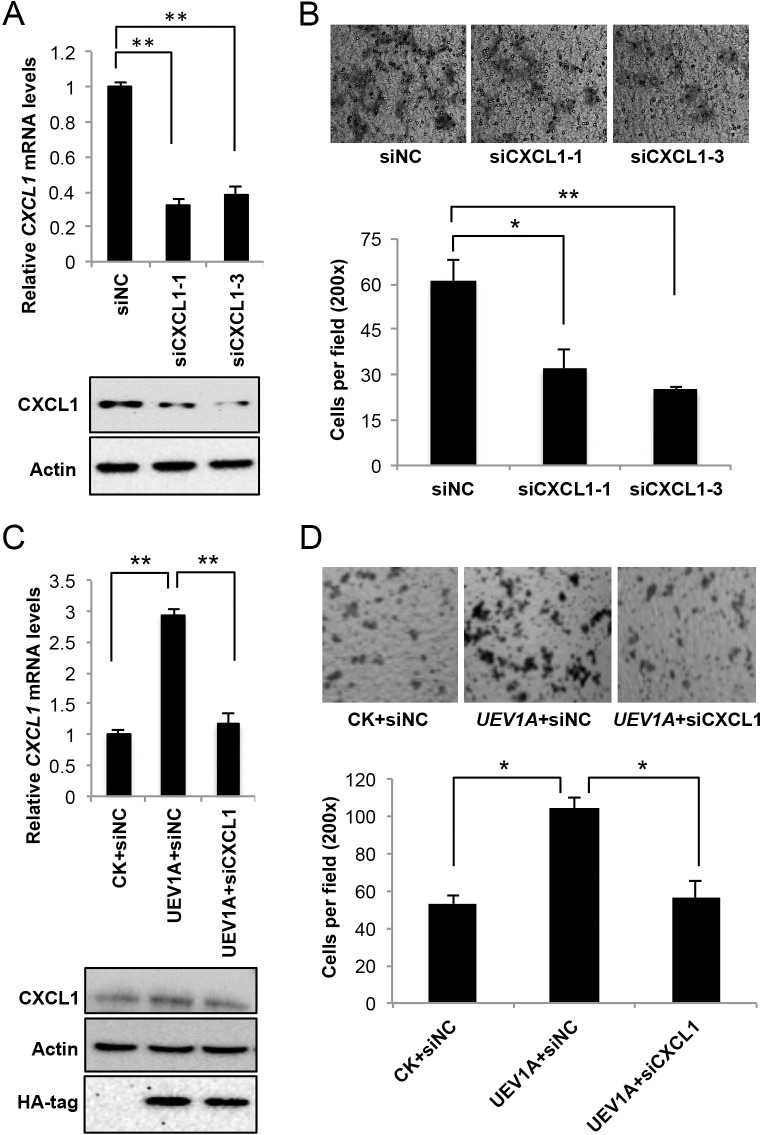
CXCL1 is a downstream effector for Uev1A-induced metastasis in HCT116 cells **(A)** The mRNA (top panel) and protein (lower panel) levels of *CXCL1* in CXCL1-depleted HCT116 cells by two independent *CXCL1* siRNAs. siNC, control siRNA. **(B)** Representative images (upper panel) and quantitative analysis (lower panel) of cell invasive ability in Matrigel-coated transwells. HCT116 cells depleted of CXCL1 were subject to the transwell assay and at least 5 random fields were counted under a light microscope at 200x magnification. **(C)** The relative transcript (upper panel) and protein (lower panel) levels of *CXCL1* in *UEV1A*-overexpressed HCT116-TR cells after CXCL1 depletion. **(D)** Representative images (upper panel) and quantitative analysis (lower panel) of cell invasive ability in Matrigel-coated transwells. *UEV1A*-overexpressed HCT116-TR cells depleted with CXCL1 were subject to the transwell assay and at least 5 random fields were counted under a light microscope at 200x magnification. ^*^ *P*<0.05, ^**^ *P*<0.01.

To ask whether CXCL1 is a critical effector for colon tumorigenesis and metastasis, we depleted CXCL1 by siRNA in HCT116 (Figure 6A) and DLD1 (Supplementary Figure 7A) cells. The depletion of CXCL1 significantly decreased the invasiveness of HCT116 and DLD1 cells as determined by a transwell assay (Figure 6B and Supplementary Figure 7B).

To ask whether Uev1A-induced *CXCL1* expression plays a critical role in colon metastasis, first, we asked if depletion of CXCL1 reverses the effect of *UEV1A* overexpression. As seen in Figure 6C, 6D, CXCL1 depletion in *UEV1A*-overexpressed HCT116 cells reduced invasiveness to a level comparable to that of control-transfected cells. Second, we overexpressed *CXCL1* in DLD1 cells about 5.4-fold to restore the *CXCL1* mRNA level close to that in *UEV1A*-overexpressed DLD1 cells (Supplementary Figure 7C). Compared with *UEV1A*-overexpressed DLD1 cells and vector control, *CXCL1* overexpression in DLD1 cells can also promote cell invasion (Supplementary Figure 7D). These results indicate that CXCL1 is a critical effector for Uev1A-induced metastasis.

### Uev1A-Ubc13 control *CXCL1* expression by regulating NF-κB

**Figure 7:**
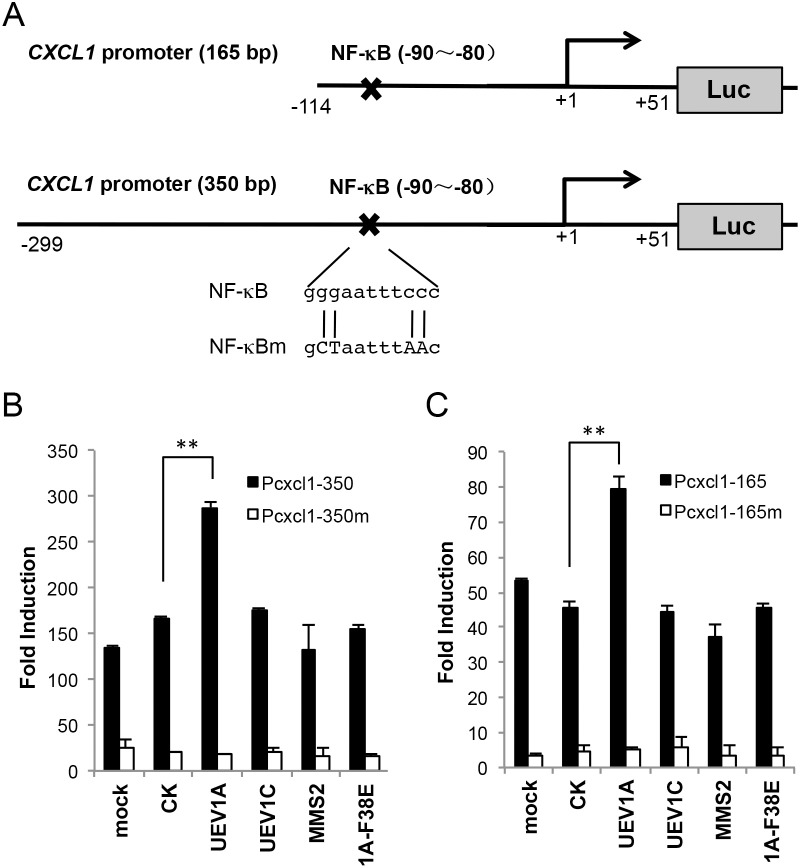
Uev1A regulates *CXCL1* expression through an NF-κB target sequence in the *CXCL1* promoter **(A)** Schematic illustration of the 165-bp (Pcxcl1-165) and 350-bp (Pcxcl1-350) *CXCL1* promoter luciferase (Luc) reporter constructs. The reported NF-κB binding site in the *CXCL1* promoter is located at −90~-80 upstream of its start codon. The mutated sequences in the P_*cxcl1*_-NF-κBm construct are also shown in capital letters. **(B)** The *P_CXCL1-350_-Luc* reporter was co-transfected to HCT116 cells with constructs that overexpressed *UEV1A*, *UEV1C, MMS2* or a mutant form of *UEV1A* (1A-F38E). 24 hours after transfection, luciferase activities were determined. The data were normalized to the activity of cells transfected with the empty vector (pGL4.2). **(C)** The *P_CXCL1-165_-Luc* reporter was co-transfected to HCT116 cells with constructs that overexpressed various *UEV* constructs and experimental conditions were as described in (B) (^**^ *P*<0.01).

The expression of the GRO gene family is regulated transcriptionally and post-transcriptionally by a number of agents, including TNFα and IL-1, through the NF-κB binding site on the 5’ regulatory region [44–46]. To ask whether Uev1A regulates *CXCL1* through NF-κB, we cloned 350-bp and 165-bp human *CXCL1* promoter sequences (Figure 7A) into pGL4.2 and then transfected them into HCT116 cells. After 40 ng/ml TNF-α induction for 2 hrs, the induced activity of each promoter construct can be detected by a luciferase assay (Supplementary Figure 8A, 8B). The promoter construct was then co-transfected with plasmids expressing *UEV1A*, *UEV1A-F38E* or an empty vector into HCT116 cells. A luciferase assay shows that *UEV1A* expression can activate the *CXCL1* promoter and that this activation relies on its interaction with Ubc13, as the Uev1A-F38E substitution completely abolished the activation (Figure 7B, 7C). The NF-κB binding sites (−90~−80) in the *CXCL1* promoter [44–46] was then mutated (Figure 7A) and this mutation completely abolished the activation of TNF-α induction (Supplementary Figure 8A, 8B) or induction by *UEV1A* expression (Figure 7B, 7C). Hence, overexpression of *UEV1A*, but not *UEV1C* or *MMS2*, can activate the wild-type *P_CXCL1_-Luc* reporter and this activation absolutely requires *UBC13* and the intact NF-κB target site.

## DISCUSSION

The current study investigates roles of *UEV1A* in tumorigenesis using a colon cancer model. It was found that, with comparable levels of ectopic expression, only *UEV1A*, but not *UEV1C* or *MMS2*, is able to promote cell migration and invasion. Similarly, overexpression of *UEV1A*, but not *UEV1C* promotes tumor growth and metastasis in a xenograft mouse model. In a reverse experiment, depletion of Uev1 in cultured colon cancer cells significantly reduces cell invasion, as well as tumor growth and metastasis, indicating that Uev1A level plays a critical role in colon cancer tumorigenesis and metastasis.

Uev1A has been reported to activate NF-κB in HepG2 cells [23] and MDA-MB-231 cells [9]. To understand the molecular mechanism by which Uev1A promotes colon cancer metastasis, we demonstrated that overexpression of *UEV1A*, but not *UEV1C* or *MMS2*, is able to promote IκBα phorsphorylation and NF-κB translocation into the nucleus, and that this effect absolutely relies on its physical interaction with Ubc13. Since NF-κB is a transcription factor that regulates the expression of a large number of genes including those involved in cancer progression, we assessed the transcript levels of a panel of NF-κB target genes thought to be involved in cancer and known to be regulated by Uev1A, including *MMP1, MMP9* [9] and *BCL2* [23]. However, NF-κB activation by *UEV1A* overexpression appears to induce different NF-κB targeting genes in different types of tumor cell lines. For example, *MMP1* is a dominant factor for metastasis in MDA-MB-231 cells [9], but it is not upregulated significantly in *UEV1A*-overexpressed HCT116 colon cancer cells. In contrast, *CXCL1* and *MMP9* are upregulated significantly in *UEV1A*-overexpressed HCT116 colon cancer cells. Since depletion of MMP9 had little effect on HCT116 cell invasion in the transwell assay, our attention focused on *CXCL1*.

CXCL1 is upregulated in various tumors, such as breast cancer [37, 47], ovarian cancer [48], prostate cancer [49, 50], gastric cancer [51] and bladder cancer [52, 53]. CXCL1 is implicated in tumorigenesis, angiogenesis, invasion, metastasis and resistance to several anti-cancer chemotherapies [37, 47]. Moreover, *CXCL1* is identified as a distinctly overexpressed gene in human colon cancers by microarray analyses [54, 55] and it promotes the growth and invasion of colon cancer cells in a mouse xenograft tumor model [42, 43, 56, 57]. To ask how Uev1A regulates *CXCL1*, we used a luciferase assay to experimentally demonstrate that Uev1A can activate the wild-type *P_CXCL1_-Luc* reporter and that this activation absolutely requires the NF-κB target site located at −90 to −80 on the 5’ regulatory region of *CXCL1* [44–46].

Overall, overexpression of *UEV1A* alone in *HCT116 cells* is sufficient to activate NF-кB, which in turn upregulates *CXCL1* expression to enhance colon cancer cell metastasis, and this phenomenon appears to be true in other colon cancer cells as well. These observations suggest a potential therapeutic target in the treatment of metastatic colon cancers.

## MATERIALS AND METHODS

### Cell culture

The human colon carcinoma cell line HCT116 was obtained from the American Type Culture Collection (ATCC). Human colon carcinoma DLD1, HT29, colo201, colo320, SW620 and human normal colon CCD-33Co cells were gifts from Dr. Keith Bonham in the Saskatoon Cancer Center. HCT116, DLD1, HT29, and SW620 cells were cultured in Dulbecco’s minimum essential medium (DMEM) (Sigma); CCD-33Co cells were cultured in Eagle’s minimum essential medium (EMEM); colo201 and colo320 cells were cultured in Roswell Park Memorial Institute (RPMI) 1640 medium. All media were supplemented with 10% fetal bovine serum, 100 units/ml penicillin, and 100 μg/ml streptomycin (Invitrogen). All cells were cultured in a 5% CO_2_ atmosphere at 37°C. HCT116-TR stable cell lines were created by transfecting HCT116 cell lines with pLenti6-TR-lentivirus (Invitrogen) and selecting with 10 μg/ml blasticidin (Invitrogen).

### Plasmids and pLentivirus vector preparation

Human *UEV1A*, *UEV1C* and *MMS2* open reading frames (ORFs) were amplified and cloned into a Dox-inducible Tet-ON plasmid pcDNA4.0/TO/HA(+) as described previously [9, 10]. The 350-bp and 165-bp human *CXCL1* promoter sequences [44–46] were PCR-amplified as *Kpn*I-*Hin*dIII fragments and then cloned into the same sites of pGL4.2 (Invitrogen). The NF-кB target site was subsequently mutated by site-directed mutagenesis using a quick-exchange method (Stratagene). The sense primer for creating the NF-кB binding site mutation is 5’-CGATCTGGAACTCCGCTAATTTAACTGGCCCGGGGGCTCC-3’ (mutated sequence underlined). The mutated Ubc13-binding site (F38E) in Uev1A was designed based on a previous study with Mms2-F13E [8]. The modified sequence for *UEV1* shRNA(sc-38606-v) and negative control shRNA(sc-108080) delivered by lentiviral particles was from Santa Cruz Biotechnology, Inc. The *UEV1* shRNA is a pool of concentrated, transduction-ready viral particales containing 3 target-specific constructs that encode 19-25 nt (plus hairpin) shRNA designed to knock down the target gene expression. Negative control shRNA lentiviral particles encode a scrambled shRNA sequence that will not lead to the specific degradation of any known cellular mRNA. The lentiviral particle infection of colon cancer cells was performed following instructions of the supplier. The *CXCL1*, *MMP1* and *MMP9* siRNAs were purchased from Genepharma Co. Ltd (Shanghai, China). The sequence for *CXCL1* siRNA-1 is 5’-CCAAGAACAUC CCAAAGUGUG-3’, *CXCL1* siRNA-3 is 5’-AGTGACAAAUCCAACUGACC-3’, *MMP1* siRNA is 5’- GCGUGUGACAGUAAGCUAA-3’, *MMP9* siRNA is 5’-CGCUCAUGUACCCUAUGUA-3’ and the negative control FAM siRNA is 5’-CUCCGAACGUGUCACGU-3’.

### RNA preparation and real-time RT-PCR (qRT-PCR)

Total RNA was prepared from cultured colon cancer cells by using TRIzol reagent (Invitrogen). First-strand cDNA was synthesized from total RNA with SuperScript (Invitrogen) according to manufacturer’s instructions. qRT-PCR analysis was performed on the iQ5 cycler (Bio-Rad). The specific primer sets were as follows: *GADPH*, 5’- GAAGGTGAAGGTCGGAGTC-3’ and 5’- GAAGATGGTGATGGGATTTC-3’; *UEV1A*, 5’- GAGAGGTTCAAGCGTCTTACCTGAA-3’ and 5’-ACT GTGCCATCTCCTACTCCTTTCT-3’; *UEV1C*, 5’-GCA GCCACCACGGGCTCG-3’ and 5’- CAATTATCAT CCCTGTCCATCTTGT-3’; *MMS2*, 5’- CGCTTGTTG GAAGAACTTGA-3’ and 5’- CGGAGGAGCTTCTGG GTAT-3’; *CXCL1* 5’-CTTGCACTCGAGGTACCCACTCCCTGGTGTCAT −3’ and 5’- GGCCAGAAGCTT CCAGGAGCAGGAGCAGCAGT −3’; *MMP1* 5’- AAAT GCAGGAATTCTTTGGG-3’ and 5’-ATGGTCCACAT CTGCTCTTG-3’; *MMP9* 5’-CATCGTCATCCAGTTTGG TG-3’ and 5’- TCGAAGATGAAGGGGAAGTG-3’. The relative expression levels were calculated using the comparative cycle threshold (CT) method (2^−ΔCT^) by the CFX Manager software (Bio-Rad).

### Luciferase reporter assay

Cells were seeded in 24-well plates at a density of 1×10^5^. After 24 hrs, the cells were transfected using X-tremeGENE HP DNA Transfection Reagent (Roche). Briefly, luciferase reporter gene constructs (500 ng), pcDNA-Uevs plasmids (500 ng) and the pRL-SV40 Renilla luciferase construct (5 ng) (for normalization) were co-transfected into the wells. Cell extracts were prepared 48 hrs after transfection and the luciferase activity was measured using the Dual-Luciferase reporter assay system (Promega).

### Preparation of nuclear fraction

HCT116 and DLD1 cells were treated with 40 ng/ml TNF-α for 2 hrs. Cells were washed, scraped with PBS, and centrifuged at 3,000 rpm at 4°C. The pellet was suspended in 10 mM Tris (pH 8.0) with 1.5 mM MgCl_2_, 1 mM DTT, and 0.1% NP-40, and incubated on ice for 15 min. Nuclei were separated from cytosol by centrifugation at 12,000 rpm at 4°C for 15 min. The cytosolic supernatants were removed and the precipitated pellets were suspended in 10 mM Tris (pH 8.0) containing 100 mM NaCl and stored on ice for 30 min. After agitation for 30 min at 4°C, the lysate was centrifuged at 12,000 rpm for 15 min at 4°C, and the supernatant was collected.

### Western blot analysis

The total cell protein was extracted and protein concentration was determined as described previously [9, 10]. Cell extracts were electrophoresed in 10% or 15% SDS-PAGE gels, transferred to PVDF membrane, and incubated with specific primary antibodies. Monoclonal antibodies (mAbs) LN1 (anti-Uev1A), LN2B (anti-Uev1) and LN3 (anti-Uev1 and Mms2) were from the lab stock [10, 58]. Primary antibodies against HA (sc-7392), NF-κB p65 (sc-372), β-tublin (sc-166729), Lamin B (sc-6216), MMP1 (sc-30069), and secondary goat anti-mouse antibody IgG-HRP (sc-2005) and goat anti-rabbit IgG-HRP (sc-2004) antibody were from Santa Cruz. The P-IκBα (#2859S) and IκBα (#44D4) antibodies were from Cell Signaling Technology, anti-MMP9 (ab38898) and anti-GRO alpha/CXCL1 (ab86436) antibodies were from Abcam, and an anti-Actin antibody (BM0005) was from Boster.

### Cell invasion assays

*In vitro* invasion assays were conducted using Transwells (Corning® 3422) with 8 μm pore-size polycarbonate membrane filters in 24-well culture plates. The upper surface of the filter was coated with Matrigel (Becton Dickinson, Bedford, MA) in a volume of 50 μl per filter. The Matrigel was dried and reconstituted at 37°C into a solid gel on the filter surface. The lower surface of the filter was coated with fibronectin (20 μg/ml), vitronectin (10 μg/ml), collagen IV (50 μg/ml), or DMEM supplemented with 10% fetal bovine serum (FBS) as chemoattractants. After starving in FBS-free DMEM overnight, 2×10^4^ cells were seeded in the upper chamber. The cells were allowed to invade for 48 hrs. Cells that invaded the lower surface of the filter were counted in 5 random fields under a light microscope at 200x magnification. Experiments were conducted at least in triplicate.

### Metastasis assay in a xenograft mouse model

The experimental mouse work followed an animal care protocol approved by the University Committee on Animal Care and Supply, University of Saskatchewan. For the tumorigenesis assays, 1×10^6^ cancer cells were injected subcutaneously into the lateral flanks of 4- to 5-week-old female athymic nude mice. The palpable tumor diameters were measured once per week. Tumor length (L) and width (W) were measured with a caliper, and the volume (V) was calculated by the following equation: V=(LxW^2^)/2. For experimental metastasis assays, the mice were sacrificed 5 weeks after cell injection. Organ samples were taken after sacrifice. Quantitative analysis of the *in vivo* organ metastasis was measured by the number of metastatic organs, 5 mice for each treatment.

### Case selection

A set of histologically normal colonic mucosa (N=31), primary colon adenocarcinoma (N=46), and metastatic colon adenocarcinoma (N=56) were selected from the pathology archives of the Royal University Hospital/Saskatoon Health Region Department of Pathology and Laboratory Medicine. REB approval was obtained from the University of Saskatchewan.

### Immunohistochemistry (IHC)

Formalin-fixed colon tissues were paraffin-embedded. The slides were deparaffinized and rehydrated. Antigen retrieval was done by cooking slides in a microwave oven in 1 mM EDTA buffer at pH 9.0 at highest power. Endogenous peroxidases were blocked. The respective slides were incubated with 200 μl of the monoclonal antibodies (mAbs) LN1 (anti-Uev1A) and NF-κB (sc-372) primary antibodies at a 1/50 dilution and incubated overnight at 4°C in a hydration chamber. Secondary antibody reactions for visualization of the bound primary Ab were performed using the Envision+ with DAB (3,3’-diaminobenzidine) as chromogen (Dako Canada). For color enhancement, subsequently the slides were incubated in a 2% copper sulphate solution for 5 min. The results were scored by an experienced pathologist (ET). Uev1A and p65 were scored as either “positive” or “negative” using 10% positive cells as cut off point. In addition, the intensity of staining was recorded as “weak” (1+), “moderate” (2+), and “strong” (3+). When samples of tumors were positive for Uev1A, they were generally diffusely positive. In contrast, when positive, the percent positive cells for p65 varied from one case to another. Therefore, Histological Score (H-Score) was used to record p65 expression. Nuclear and cytoplasmic staining was recorded independently for both p65 and Uev1A, and only nuclear positivity for p65 was used for further analysis and calculations. Subsequently, the H-score results were categorized for presentation purposes as follows: 0 (H-score <10), 1 (H-score = 10 – 100), 2 (H-score 101 – 200), and 3 (H-score = 201 – 300). Results of the pathology readout were analyzed by SPSS statistical software by using the Spearman Correlation Test to compare levels of p65 and Uev1A expression and the Pearson Chi-Square Test to determine associations between Uev1A expression and diagnostic group. Results with *P*-values <0.05 were considered to be significant. Microscopic images were captured by a SPOT digital camera mounted in a light microscope.

### Statistical analysis

The statistical significance of differential findings between the experimental and control groups was determined by a Student’s t-test as implemented by Excel 2010 (Microsoft), and *P* <0.05 was considered significant.

## SUPPLEMENTARY MATERIALS FIGURES AND TABLES


