# Supplementary material for: Uev1A-Ubc13 promotes colorectal cancer metastasis through regulating CXCL1 expression via NF-кB activation
Source: Oncotarget. 2018 Mar 23;9(22):15952–67. doi: 10.18632/oncotarget.24640 (PMC5882310; doi:10.18632/oncotarget.24640)
Supplement: Supplementary file 1 [file oncotarget-09-15952-s001.pdf]

Uev1A-Ubc13 promotes colorectal cancer metastasis through regulating *CXCL1* expression via NF-κB activation

SUPPLEMENTARY MATERIALS

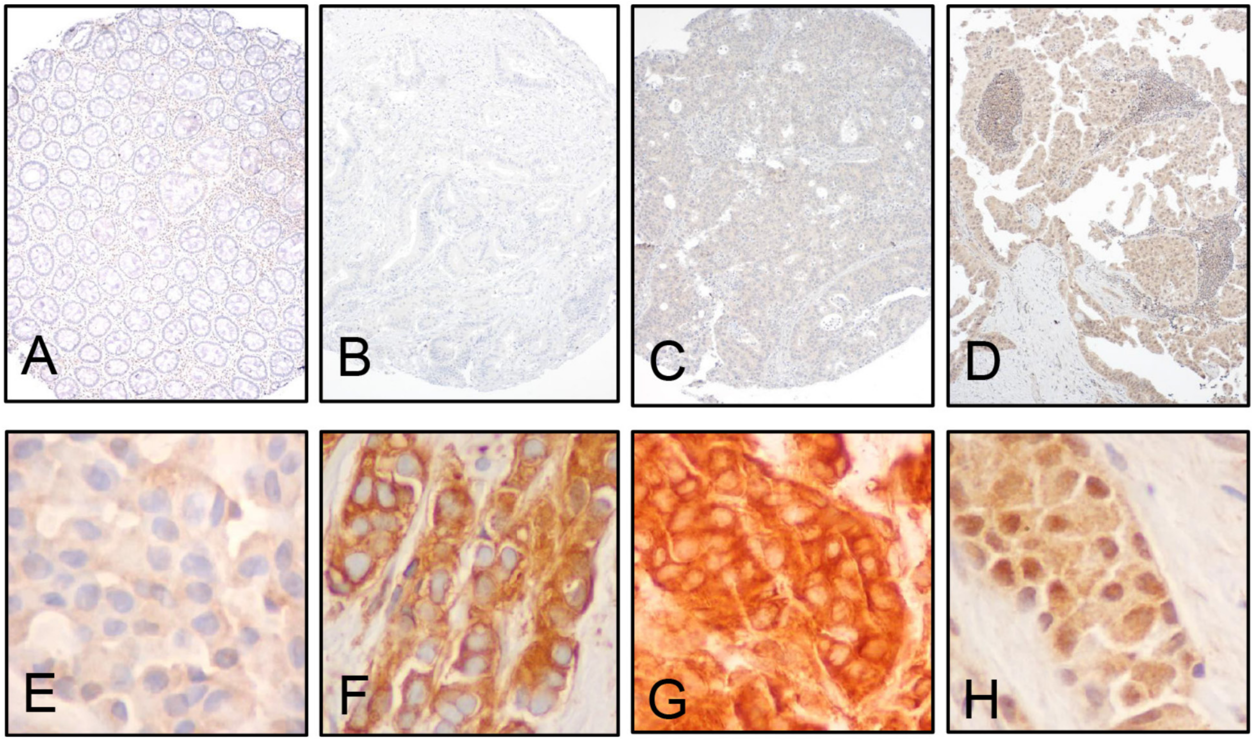

Supplementary Figure 1: IHC score of colon tissue samples. **(A-D)** IHC with the LN1 anti-Uev1A monoclonal antibody. (A) A normal colon mucosa showing no staining for Uev1A in the epithelial cells and weak staining in lamina propria lymphocytes (B) Primary adenocarcinoma negative for Uev1A. (C) Primary adenocarcinoma with weak diffuse cytoplasmic staining for Uev1A, which was scored 1+ for intensity of staining and 100% for percent positive cells. (D) Metastatic adenocarcinoma positive for Uev1A, which was scored 3+ for intensity of staining and 100% for percent positive cells. **(E-H)** IHC with and the anti-p65 antibody. Only nuclear staining was interpreted as "positive for p65". (E) There is no definite nuclear staining for p65 and cytoplasmic staining is also generally absent (H-score <10%, category "0"). (F) Weak to moderate nuclear staining was present in rare cells, while cytoplasmic staining was strong (H-score 58, category "1"). (G) Weak to moderate nuclear staining was present in all cells with strong cytoplasmic staining (H-score 144, category "2"). (H) Moderate to strong nuclear staining was detected in most cells with only weak cytoplasmic staining (H-score 239, category "3").

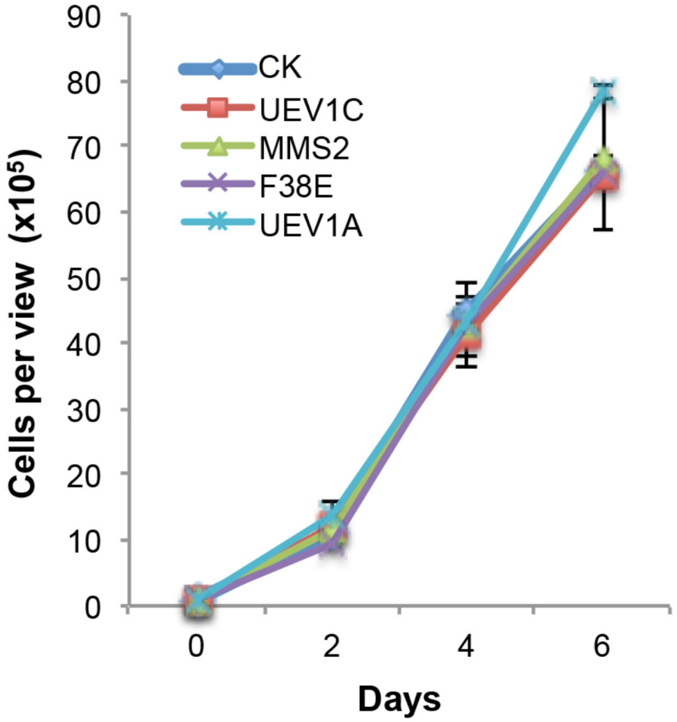

Supplementary Figure 2: Growth curve of HCT116-TR cells expressing ectopic *UEV* genes. Viable cells were counted at the given time intervals and expressed as number of cells per view. The cells carrying an empty vector served as control (CK). Results are the average of three independent experiments with standard deviations.

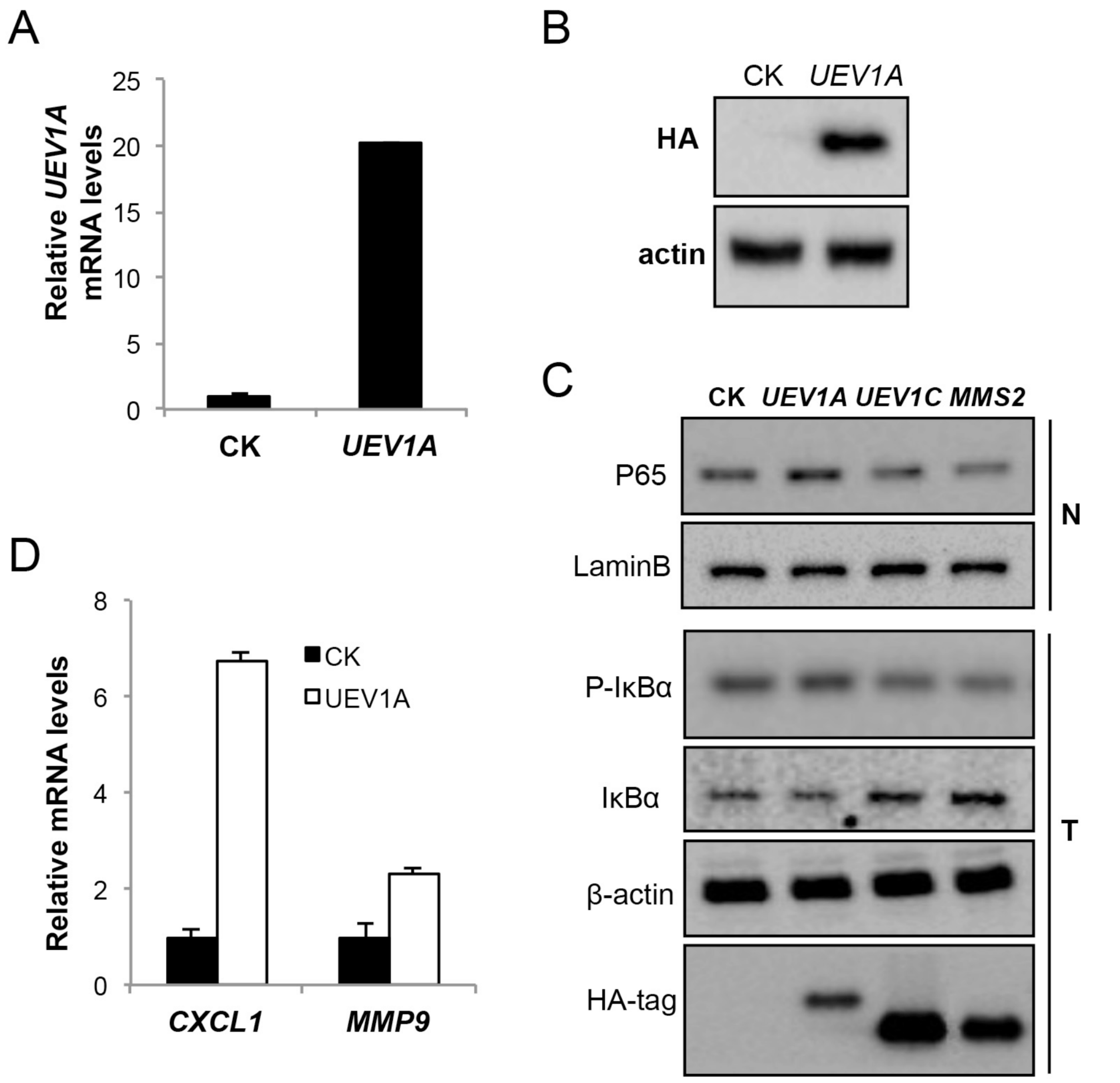

Supplementary Figure 3: Effects of *UEV1A* overexpression in DLD1 cells. pcDNA4.0/TO/HA(+) vector expressing *UEV1A* or vector only (CK) was transfected into DLD1 cells. The level of ectopic gene expression was monitored by qRT-PCR **(A)** and western blot against an anti-HA antibody **(B)**. **(C)** NF-κB activation in *UEV*-overexpressing cells. pcDNA4.0/TO/HA(+) vector expressing *UEV1A*, *UEV1C* or *MMS2* was transfected into DLD1 cells. Nuclear (N) or whole-cell (T) extracts were prepared, and equal amounts of protein were separated by SDS-PAGE gel, followed by western blotting analysis using an anti-p65 antibody to measure NF-κB nuclear enrichment, an anti-P(S32)-IκBα antibody and an anti-IκBα antibody to assess the degree of IκBα phosphorylation and its degradation and release of NF-κB into the nucleus. **(D)** Elevated *CXCL1* and *MMP9* mRNA levels in *UEV1A*-overexpressed DLD1 cells as determined by qRT-PCR.

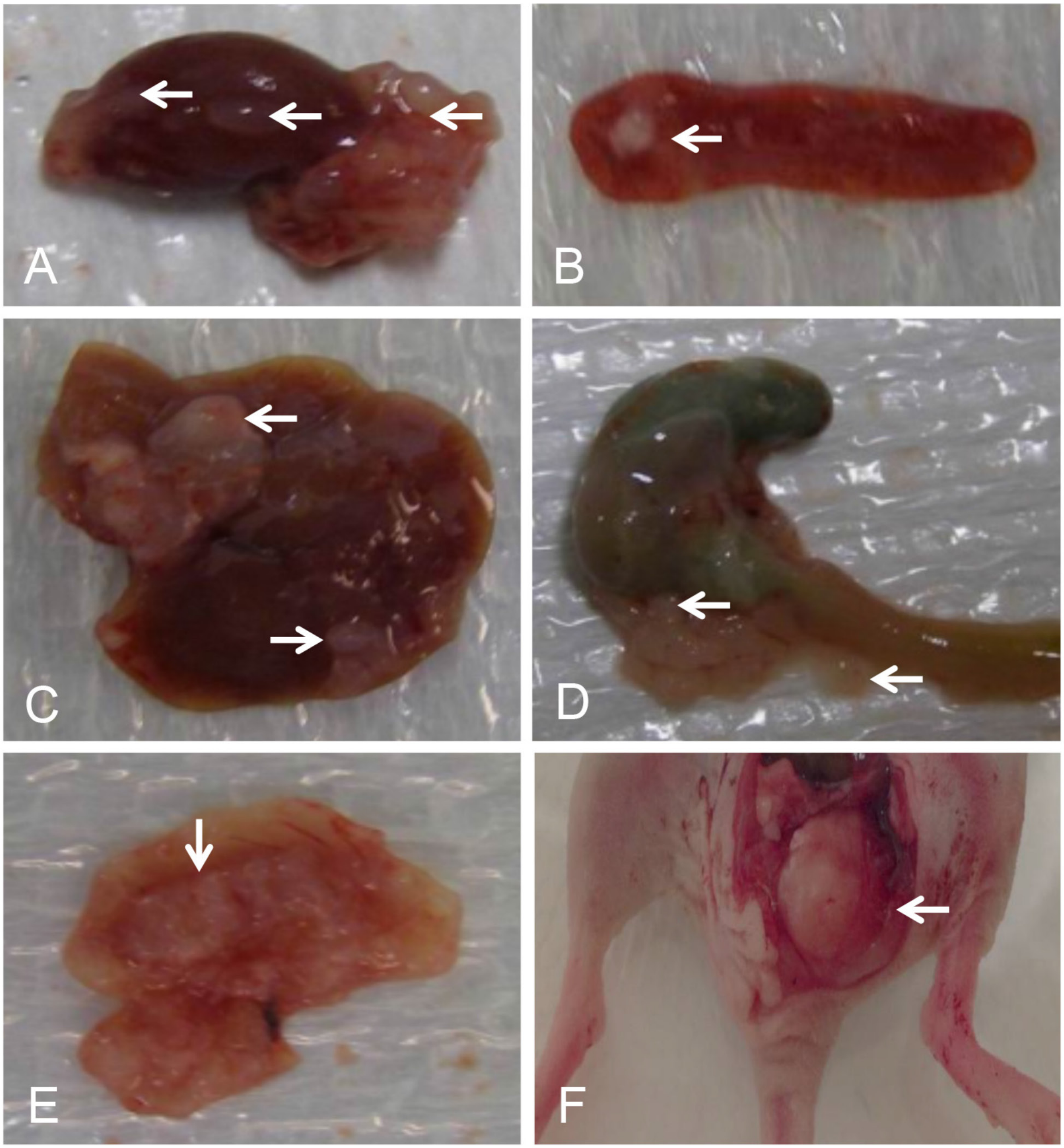

Supplementary Figure 4: *In vivo* metastasis assays using a xenograft mouse model. 1×10^6 HCT116-TR cells collected from each treatment were injected into the lateral flanks of 4- to 5-week-old female athymic nude mice. Five weeks after injection, organ samples were taken after sacrifice. Quantitative analysis of the *in vivo* organ metastasis as measured by the number of metastatic organs (n = 5). The macroscopic view of the typical metastatic lesions in *UEV1A* overexpressed group is shown by red arrows. **(A)** Kidney. **(B)** Spleen. **(C)** Liver. **(D)** Stomach and esophagus. **(E)** Diaphragm. **(F)** Other.

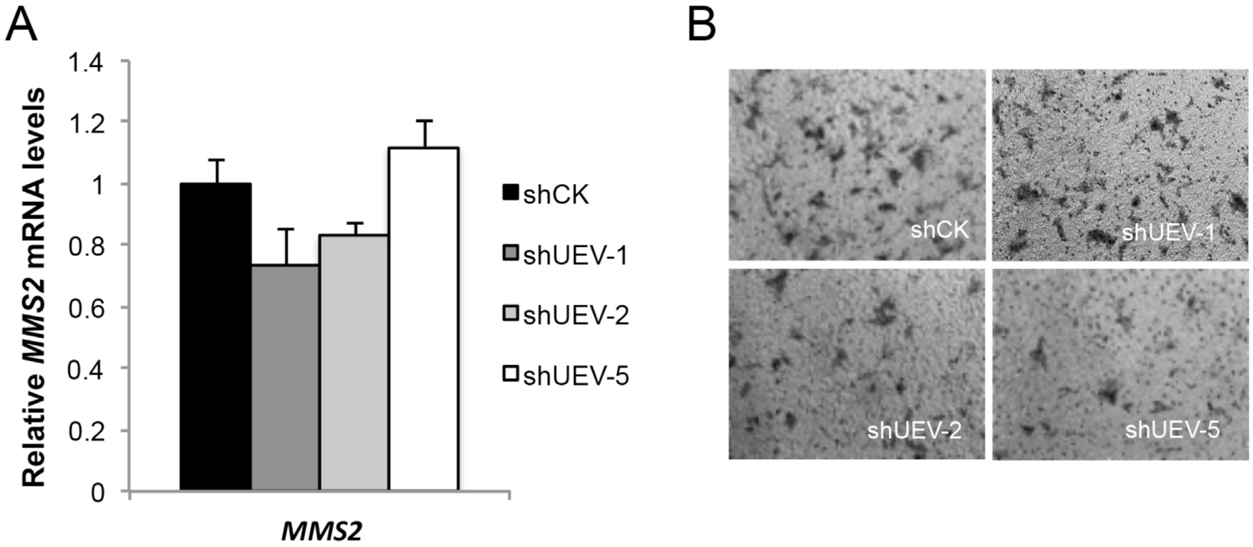

Supplementary Figure 5: Effects of Uev1 depletion on *MMS2* expression and cell invasion in HCT116 cells. (A) HCT116 cells were transfected with shRNA lentiviral particles either against *UEV1* (sh*UEV1*) or non-specific target (shCK). The transcript levels of *MMS2* in three independent shRNA lines were determined by qRT-PCR. **(B)** Representative images of cell invasion assay with Matrigel-coated transwells in non-specific target (shCK) and three independent stable shUEV1 lines.

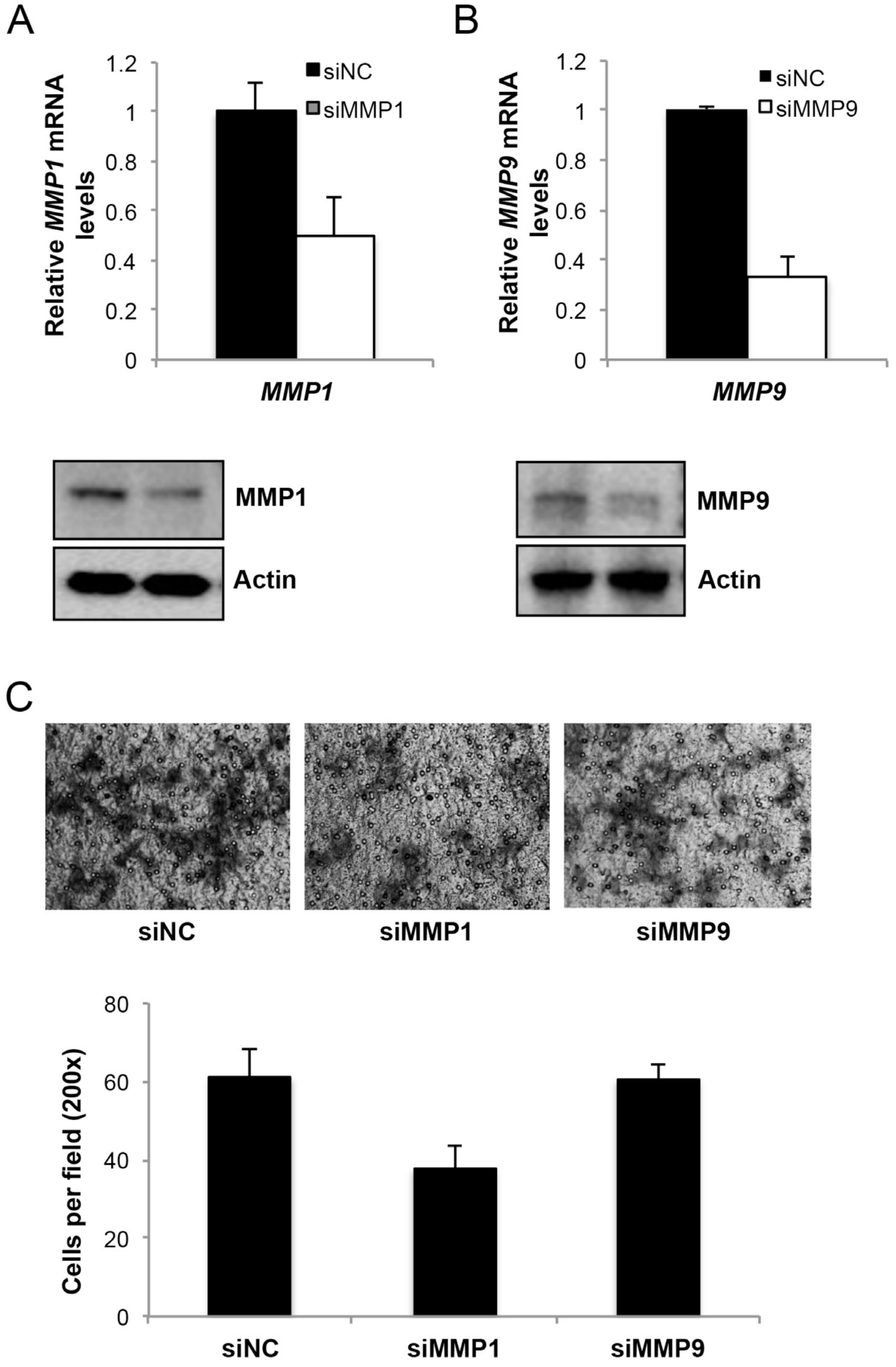

Supplementary Figure 6: Effects of MMP1 and MMP9 depletion on HCT116 cell invasion. **(A)** The *MMP1* mRNA (upper panel) and MMP1 protein (lower panel) levels in *MMP1*-depleted HCT116 cells. **(B)** The *MMP9* mRNA (upper panel) and MMP9 protein (lower panel) levels in *MMP9*-depleted HCT116 cells. **(C)** Cell invasive ability in Matrigel-coated transwells after MMP1 or MMP9 depletion. Upper panel, representative images; lower panel, quantitative analysis. At least 5 random fields were counted under a light microscope at 200x magnification. siNC, control siRNA.

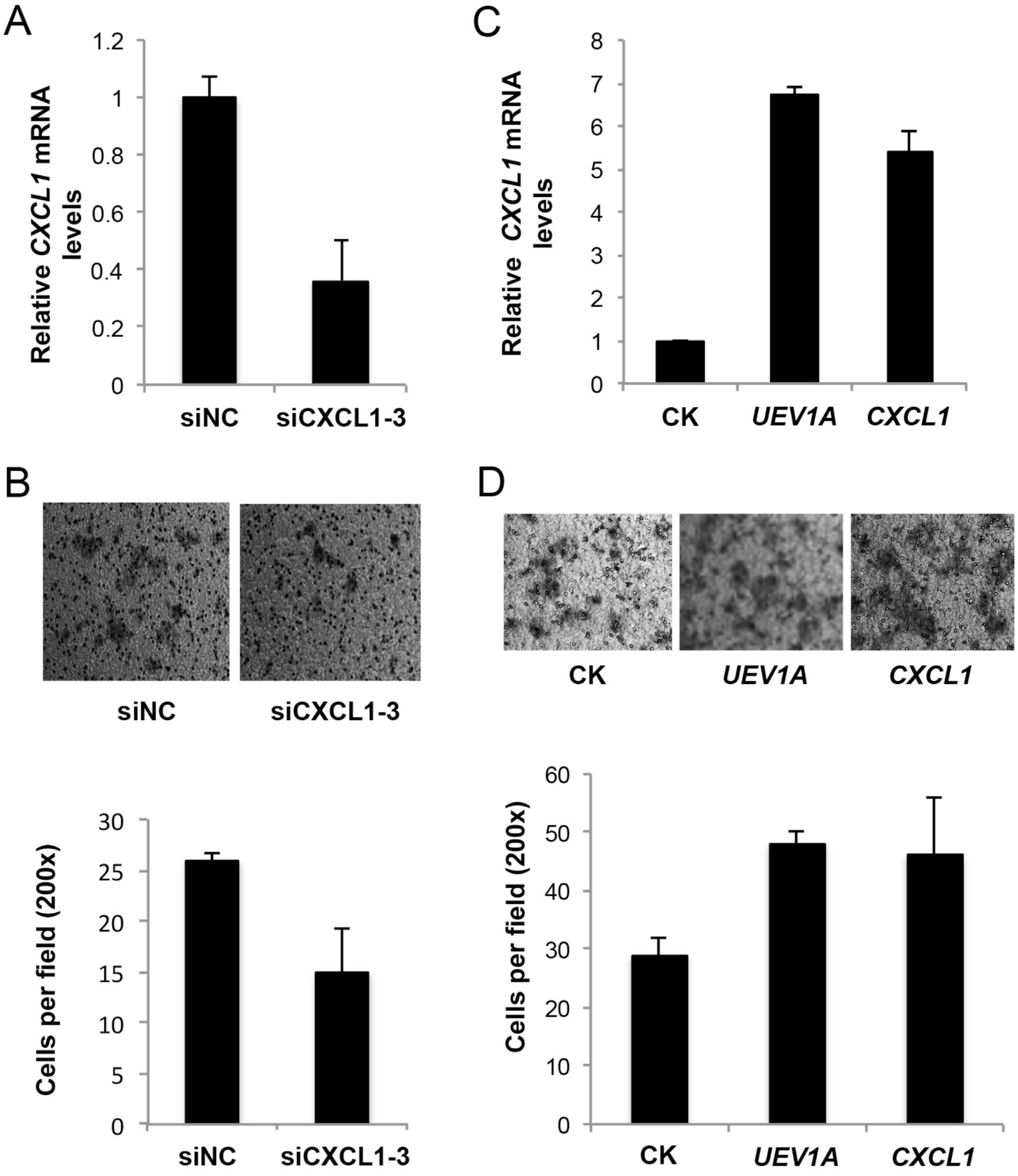

Supplementary Figure 7: Critical roles of CXCL1 in Uev1A-mediated metastasis in DLD1 cells. **(A)** The *CXCL1* mRNA level in DLD1 cells depleted of *CXCL1* by *CXCL1* siRNA as determined by qRT-PCR. **(B)** Cell invasive ability in Matrigel-coated transwells after CXCL1 depletion. Upper panel, representative images; lower panel, quantitative analysis of at least 5 random fields were counted under a light microscope at 200x magnification. **(C)** pcDNA4.0/TO/HA(+) vector expressing *UEV1A* or *CXCL1* was transfected into DLD1 cells. The level of ectopic gene expression was monitored by qRT-PCR. **(D)** Cell invasive ability in Matrigel-coated transwells after *UEV1A* or *CXCL1* overexpression. Upper panel, representative images; lower panel, quantitative analysis of at least 5 random fields were counted under a light microscope at 200x magnification.

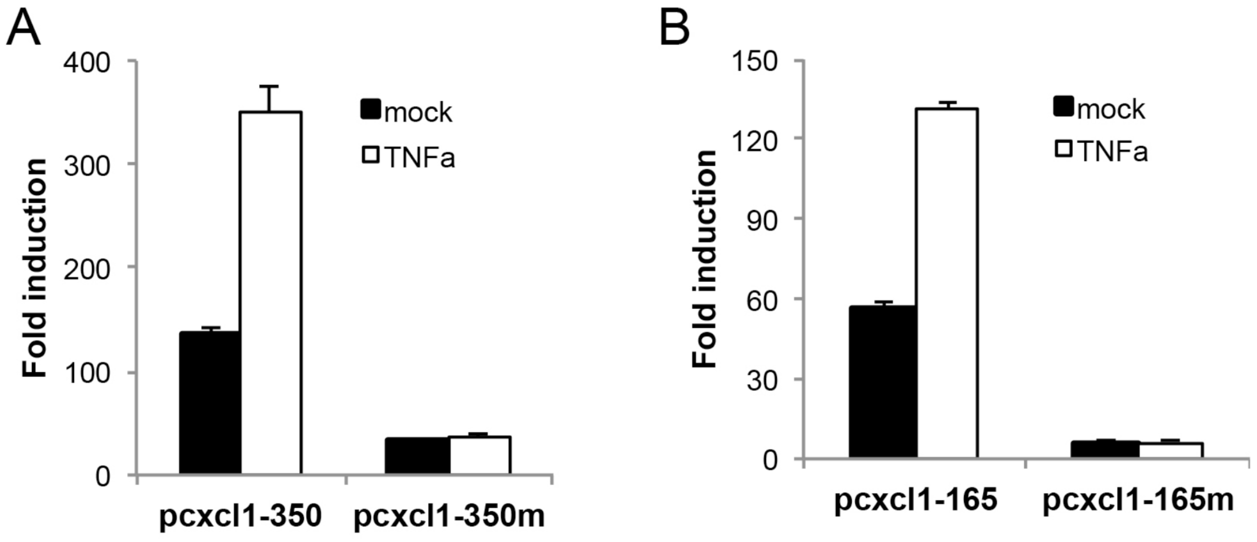

Supplementary Figure 8: Relative levels of TNFα-induced *P_{CXCL1}-Luc* expression. The *P_{CXCL1}-Luc* reporters or their mutant construct were transfected to HCT116 cells. After 24 hrs, cells were treated with or without TNFα for 30 min. Luciferase activities were determined. **(A)** *$P_{CXCL1\text{-}350}$-Luc* **(B)** *$P_{CXCL1\text{-}165}$-Luc*.
